# Disseminated cryptococcosis in Crohn’s disease: a case report

**DOI:** 10.1186/s12879-018-3553-3

**Published:** 2018-12-04

**Authors:** Natthakit Chavapradit, Nasikarn Angkasekwinai

**Affiliations:** Division of Infectious Diseases and Tropical Medicine, Department of Medicine, Faculty of Medicine Siriraj Hospital, Mahidol University, 2 Wanglang Road, Bangkoknoi, Bangkok, 10700 Thailand

**Keywords:** *Cryptococcus neoformans*, Crohn’s disease, Gastrointestinal cryptococcosis

## Abstract

**Background:**

Gastrointestinal (GI) cryptococcosis is rarely reported. Most cases were diagnosed during evaluation of comorbid conditions, incidental findings, or postmortem. Here, we present a case of Crohn’s disease with gastrointestinal cryptococcosis that resembled exacerbation of Crohn’s disease.

**Case presentation:**

A 64-year-old woman with Crohn’s disease (CD) was referred to Siriraj Hospital due to worsening of abdominal pain and watery diarrhea for 2 weeks. The dose of immunosuppressive agents was increased for presumed exacerbation of CD. Pathologic examination of tissue obtained from polypoid mass at ileocecal valve and multiple clean-based ulcers at cecum revealed active ileitis and colitis with multiple round shape organisms with capsule, which was compatible with *Cryptococcu*s species. Disseminated cryptococcosis was diagnosed due to gastrointestinal involvement and presumed pulmonary involvement regarding the presence of an oval-shaped cavitary lesion on chest X-ray and computed tomography of the lung. Patient was successfully treated with amphotericin B followed by fluconazole with satisfactory result.

**Conclusion:**

Early diagnosis of gastrointestinal cryptococcosis in Crohn’s disease is difficult due to the lack of specific symptoms and sign or mimicking an exacerbation of Crohn’s disease. Seeking for other site of involvement in disseminated cryptococcosis including lung or central nervous system as well as detection of serum cryptococcal antigen would be helpful for early diagnosis and management.

## Introduction

Cryptococcosis is a significant opportunistic infection in non-HIV immunocompromised individuals, including those with solid organ transplantation, malignancy, diabetes, rheumatologic disease, or chronic liver disease [1–3]. Clinical presentation of cryptococcosis varies from asymptomatic to life-threatening central nervous system (CNS) involvement. Meningitis was reported to be the predominate manifestation of cryptococcosis among HIV-infected patients [1], with pulmonary or skin and soft tissue involvement found to be more prevalent among non-HIV immunocompromised individuals [4]. Gastrointestinal (GI) cryptococcosis is rarely reported, both in disseminated disease and in cases with localized involvement [5]. Here, we report a case of Crohn’s disease with disseminated cryptococcosis due to GI and pulmonary involvement.

## Case presentation

A 64-year-old woman with a history of Crohn’s disease (CD) was referred to Siriraj Hospital located in Bangkok, Thailand in June 2017 due to worsening of abdominal pain and watery diarrhea for 2 weeks. She was diagnosed with CD in 2010 after she presented with abdominal pain and palpable mass at the right lower quadrant (RLQ) of her abdomen. She was treated successfully with prednisolone 40 mg/day, azathioprine 50 mg/day, and mesalazine 2400 mg/day. Her disease was then controlled with prednisolone 5 mg/day and the same dose of azathioprine and mesalazine. One year after her CD diagnosis, she developed severe abdominal pain and diarrhea. A colonoscopy was performed, which showed inflamed mucosa and a clean-based ulcer at the cecum. Biopsy revealed an intracytoplasmic inclusion body, which is consistent with cytomegalovirus (CMV) colitis. Her symptoms gradually subsided after 3 weeks treatment of intravenous ganciclovir 5 mg/kg twice daily. Two years after her CD diagnosis, she began experiencing recurrent abdominal pain. Abdominal computed tomography (CT) and colonoscopic findings revealed a large polypoid mass at the ascending colon with partial colonic obstruction that was suspected to be caused by active CD. She was managed conservatively and was discharged home with prednisolone 30 mg/day, azathioprine 150 mg/day, and mesalazine 2400 mg/day after making substantial improvement. Even though she remained stable for about 4 years after her most recent discharge, she still required prednisolone 5 mg/day, azathioprine 150 mg/day, and mesalazine 2400 mg/day to control her disease activity. Her last colonoscopy with biopsy, which was performed 6 months earlier to monitor her disease activity, revealed a shallow 1 cm^2^ ulcer that was covered with white exudate at the cecum, and multiple polypoid masses at the ileocecal (IC) valve. The histopathologic findings revealed a chronic ulcer with marked active inflammation, a hyperplastic polyp with chronic ileitis, and no viral inclusion, dysplasia, or malignancy. Eight weeks ago, she started complaining of intermittent cramping at the RLQ of her abdomen. The dose of azathioprine was increased to 175 mg/day for presumed exacerbation of CD; however, her pain worsened, and she eventually developed a low-grade fever and watery diarrhea for 2 weeks before admission. She denied having any pulmonary symptoms. On examination, her body temperature was 38.6 °C and she had abdominal tenderness and a palpable mass at the RLQ of her abdomen. All other examination findings were unremarkable.

**Fig. 1:**
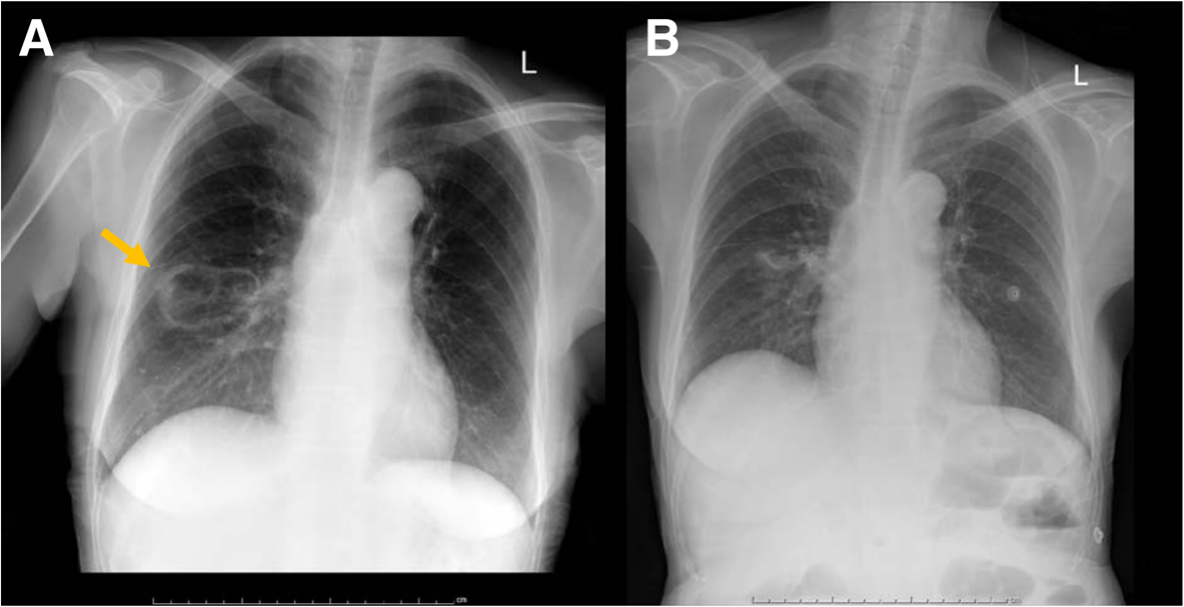
Chest radiograph before and after antifungal treatment. **a** Chest radiograph before treatment with oval-shaped cavitary lesion at the right lower lung field. **b** Chest radiograph showing lesion improvement at 3 months after start of treatment

**Fig. 2:**
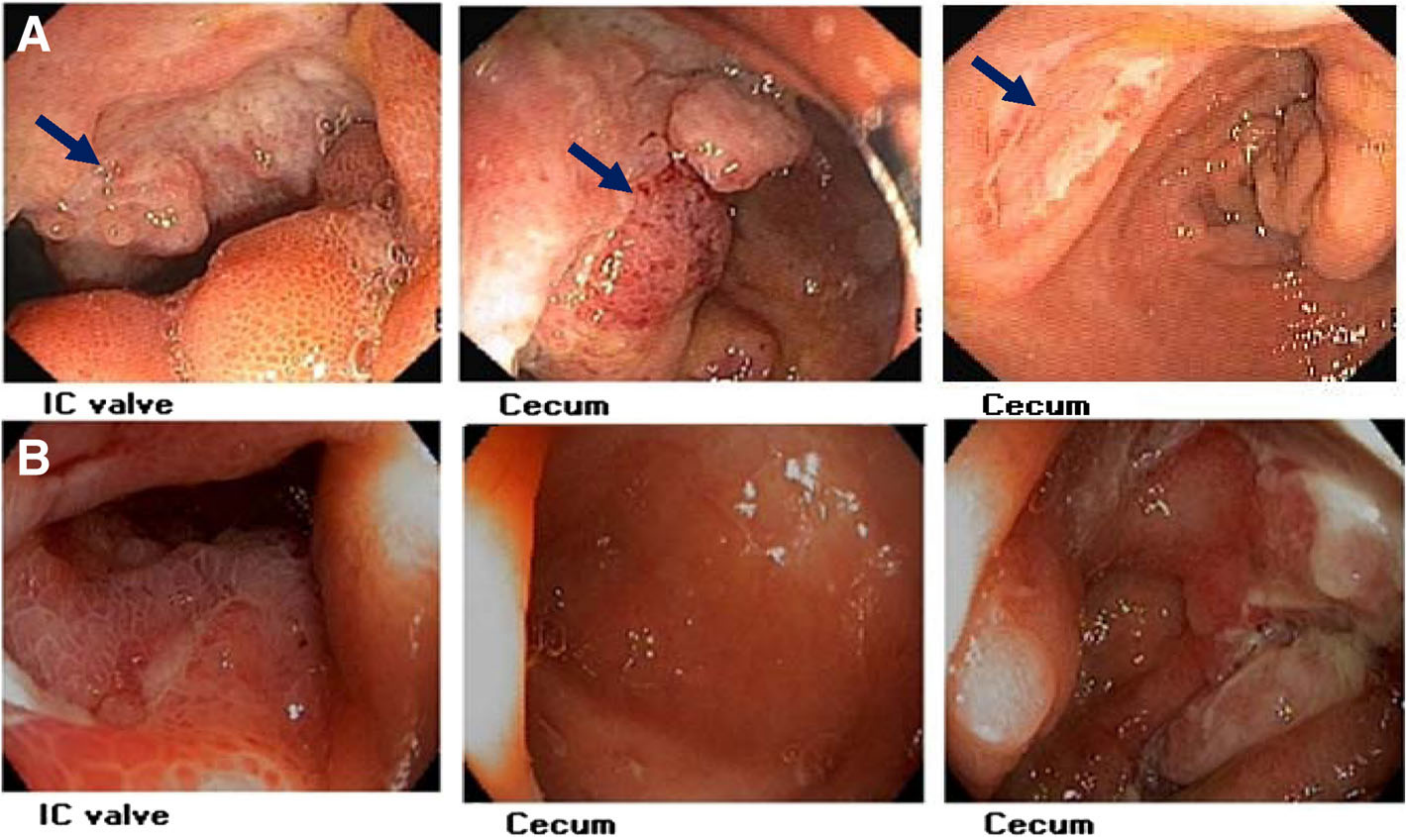
Colonoscopic finding before and after antifungal treatment. **a** Colonoscopic finding before treatment reveal polypoid mass at IC valve with multiple clean-based ulcer at cecum. **b** Colonoscopic finding 10 weeks after treatment show improvement of polypoid mass like lesion at IC valve and ulcer at cecum

**Fig. 3:**
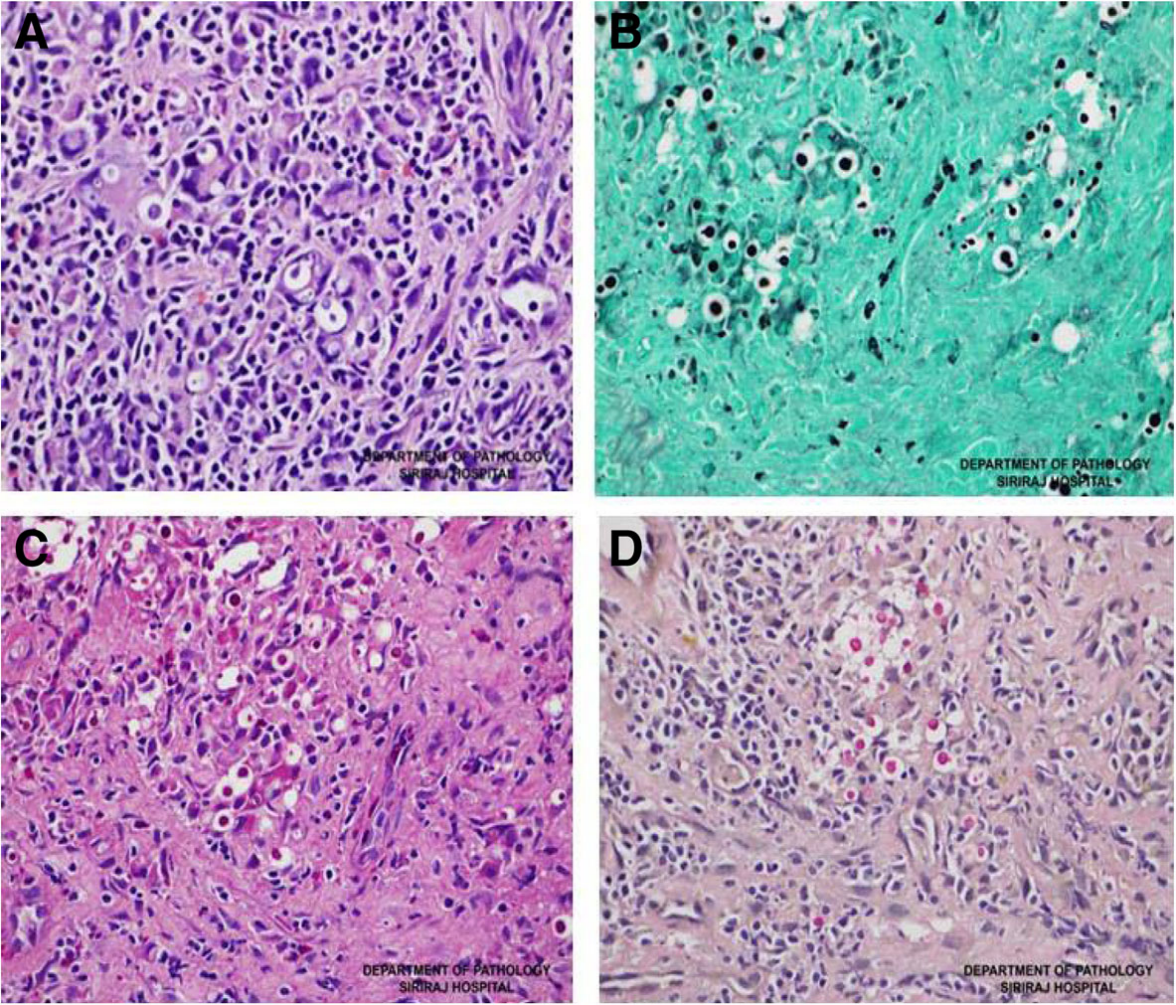
Pathologic examination of the tissue biopsy for each special stain demonstrated active ileitis and colitis with encapsulated, round-shape yeast form, which is consistent with cryptococcosis. **a** hematoxylin and eosin (H&E) stain, **b** Grocott-Gomori’s methenamine silver (GMS) stain, **c** periodic acid-Schiff (PAS) stain, and **d** mucicarmine stain

Laboratory investigations revealed a white blood cell (WBC) count of 2010 cells/mm^3^ with 90.5% neutrophils and 8.5% lymphocytes, and a hemoglobin level of 8.2 g/dl. Chest X-ray showed an oval-shaped cavitary lesion at the right lower lung (Fig. 1a). A CT scan of the chest revealed a large cavitary lesion (5.4 × 3.2 cm) with irregular wall thickening at the right lower lobe, and an adjacent focal centrilobular nodule with tree-in-bud pattern. A CT scan of the abdomen revealed chronic wall thickening at the cecum, IC valve, and terminal ilium, with submucosal edema and pericolic fat stranding. The patient underwent colonoscopy and biopsy, and a polypoid mass was observed at the IC valve with multiple clean-based ulcers at the cecum (Fig. 2a). Since the initial diagnosis was active CD, the tissue was sent only for histopathology. No fresh tissue was submitted for fungal culture. Pathologic examination of the tissue biopsy revealed active ileitis and colitis with multiple round shape organisms. The capsules of the observed organisms could be visualized by hematoxylin and eosin (H&E), periodic acid-Schiff (PAS), Grocott-Gomori’s methenamine silver (GMS) staining, and mucicarmine stain, which is compatible with *Cryptococcu*s spp. (Fig. 3). Even though serum cryptococcal antigen was positive with a titer of 1:128, blood, bone marrow, and cerebrospinal fluid cultures for fungus were all negative.

The final diagnosis was disseminated cryptococcosis due to gastrointestinal and presumed pulmonary involvement. Initial induction therapy with amphotericin B deoxycholate (AmBD) 0.7 mg/kg per day was given for 6 weeks, followed by consolidation therapy with fluconazole 800 mg/day for 8 weeks. After consolidation, maintenance treatment with fluconazole 200 mg/day was prescribed for up to 1 year. After diagnosis of disseminated cryptococcosis, the immunosuppressive agents were adjusted as follow: discontinued azathioprine, decreased the dose of prednisolone to 15 mg/day, and maintained mesalazine at 2400 mg/day. Her clinical status was gradually improved, and the colonoscopy that was performed at 10 weeks after the start of treatment showing marked improvement in the polypoid mass-like lesion at the cecum (Fig. 2b). Even though the diagnosis of pulmonary cryptococcosis was not confirmed by pathology or microbiology, her chest X-ray the 3-month follow-up showed marked improvement (Fig. 1b).

## Discussion

Cryptococcosis is uncommon and difficult to diagnose in non-HIV infected immunocompromised patients, and this leads to delayed treatment and higher rates of mortality than the rates observed in HIV-infected patients [3]. Gastrointestinal (GI) cryptococcosis is even more rarely reported condition. Our review of the literature revealed only a small number of reported cases of GI cryptococcosis. The majority of cases were asymptomatic and were diagnosed during evaluation of comorbid conditions, by incidental findings, or at postmortem [6]. A retrospective review of 24 autopsy cases with pulmonary or disseminated cryptococcosis revealed a 33% prevalence of GI involvement [5]. A review of 30 cases of GI cryptococcosis that were published during 1951 to 2008 found that 87% of patients were immunocompromised, with most of those having HIV infection [6]. While almost 90% of the immunocompromised patients had disseminated disease with GI involvement, none of the immunocompetent patients had disseminated disease. Moreover, even though GI infection can develop at any level of the digestive tract, the colon was the most frequent site of infection [5].

Patients with inflammatory bowel disease (IBD) are at increased risk for developing opportunistic infections due to factors that include receiving immunosuppressive and immunomodulatory therapy [particularly anti-tumor necrosis factor-α (anti-TNF-α) agents], severity of disease, and malnutrition [7–10]. A systematic review of 1524 patients with IBD and coexisting fungal infection reported that most fungal infections were caused by *Candida* spp. or *Histoplasma capsulatum*. The most frequently observed sites of infection were the respiratory and GI tracts. However, most of the reviewed studies were conducted in North America and Europe [11]. More studies are needed to understand the epidemiology of fungal infection in patients with IBD in Southeast Asia.

There is no direct relationship between CD and cryptococcosis. Among 9 reported cases of cryptococcosis in CD patients, 6 had pulmonary cryptococcosis [12–14], and 2 cases had GI cryptococcosis as part of their disseminated disease during anti-TNF-α therapy [6, 15]. One case was reported in an apparently healthy 26-year-old woman who presented with abdominal pain, weight loss, diarrhea, and melena, and who was eventually diagnosed with coexisting disseminated cryptococcosis and CD [16]. GI cryptococcosis in patients with CD may be promoted by mucosal injury that facilitates pathogen penetration. Similar to previous findings, our case had disseminated disease with GI and probable pulmonary involvement. Even though our patient did not receive anti-TNF-α therapy, she has been on corticosteroid and immunosuppressive agents since her CD diagnosis, which rendered her more vulnerable to various opportunistic infections.

According to the practice guidelines for the management of cryptococal diseases, non-CNS, non-severe pulmonary, localized cryptococcal infection can be treated with fluconazole alone. However, patients with disseminated disease (involvement at ≥2 noncontiguous sites) in non-HIV and non-transplant patients should be treated as CNS disease, as follows: amphotericin B deoxycholate (AmBD) 0.7–1 mg/kg per day for at least 6 weeks or AmBD 0.7 mg/kg per day plus flucytosine (100 mg/kg/day) for at least 4 weeks, followed by consolidation therapy with fluconazole (400–800 mg/day orally) for 8 weeks, and maintenance therapy with fluconazole (200 mg/day) for a total duration of 6–12 months [17]. Since flucytosine is not available in Thailand, fluconazole was used in combination with AmBD with satisfactory results [18].

Early diagnosis of GI cryptococcosis in CD is difficult due to the lack of specific symptoms and signs, and the fact that GI cryptococcosis tends to mimic exacerbation of CD. Investigation for other common sites of involvement in disseminated cryptococcosis (including lung and CNS), as well as detection of serum cryptococcal antigen, would help to facilitate early diagnosis, appropriate management, and more favorable outcomes.

## Abbreviations

AmBD: Amphotericin B deoxycholate
anti-TNF-α: Anti-tumor necrosis factor-α
CD: Crohn’s disease
CMV: Cytomegalovirus
CNS: Central nervous system
CT: Computed tomography
GI: Gastrointestinal
GMS: Grocott-Gomori’s methenamine silver
H&E: Hematoxylin and eosin
HIV: Human immunodeficiency virus
IBD: Inflammatory bowel disease
PAS: Periodic acid-Schiff
RLQ: Right lower quadrant
WBC: White blood cell
